# Cognitive Dysfunction Precedes the Onset of Motor Symptoms in the MitoPark Mouse Model of Parkinson’s Disease

**DOI:** 10.1371/journal.pone.0071341

**Published:** 2013-08-15

**Authors:** Xiuhua Li, Laney Redus, Cang Chen, Paul A. Martinez, Randy Strong, Senlin Li, Jason C. O’Connor

**Affiliations:** 1 Department of Pharmacology, The University of Texas Health Science Center, San Antonio, Texas, United States of America; 2 Department of Medicine, The University of Texas Health Science Center, San Antonio, Texas, United States of America; 3 Center for Biomedical Neuroscience, The University of Texas Health Science Center, San Antonio, Texas, United States of America; 4 Mood Disorders Translational Research Core, The University of Texas Health Science Center, San Antonio, Texas, United States of America; 5 Geriatric Research, Education and Clinical Center, South Texas Veterans Health Care System, San Antonio, Texas, United States of America; 6 Barshop Institute for Longevity and Aging Studies, San Antonio, Texas, United States of America; 7 Department of Neurology, Shandong Provincial Qianfoshan Hospital, Shandong University, Jinan, P.R. China; University of Chicago, United States of America

## Abstract

Parkinson’s disease (PD) is a neurodegenerative disorder primarily characterized by progressive loss of dopamine neurons, leading to loss of motor coordination. However, PD is associated with a high rate of non-motor neuropsychiatric comorbities that often develop before the onset of movement symptoms. The MitoPark transgenic mouse model is the first to recapitulate the cardinal clinical features, namely progressive neurodegeneration and death of neurons, loss of motor function and therapeutic response to L-DOPA. To investigate whether MitoPark mice exhibit early onset of cognitive impairment, a non-motor neuropsychiatric comorbidity, we measured performance on a spatial learning and memory task before (∼8 weeks) or after (∼20 weeks) the onset of locomotor decline in MitoPark mice or in littermate controls. Consistent with previous studies, we established that a progressive loss of spontaneous locomotor activity began at 12 weeks of age, which was followed by progressive loss of body weight beginning at 16–20 weeks. Spatial learning and memory was measured using the Barnes Maze. By 20 weeks of age, MitoPark mice displayed a substantial reduction in overall locomotor activity that impaired their ability to perform the task. However, in the 8-week-old mice, locomotor activity was no different between genotypes, yet MitoPark mice took longer, traveled further and committed more errors than same age control mice, while learning to successfully navigate the maze. The modest between-day learning deficit of MitoPark mice was characterized by impaired within-day learning during the first two days of testing. No difference was observed between genotypes during probe trials conducted one or twelve days after the final acquisition test. Additionally, 8-week-old MitoPark mice exhibited impaired novel object recognition when compared to control mice. Together, these data establish that mild cognitive impairment precedes the loss of motor function in a novel rodent model of PD, which may provide unique opportunities for therapeutic development.

## Introduction

Parkinson’s disease (PD) is the second most common progressive neurodegenerative disorder and primarily affects the dopamine (DA) neurons of the substantia nigra pars compacta (SNpc). Like many neurodegenerative diseases, the cause of PD is not known, but a combination of genetic and environmental factors is almost certainly involved. Characterized by motor impairments due to a loss of nigrostriatal dopaminergic neurons, PD is not diagnosed until the onset of motor deficits. However, there are many non-motor symptoms associated with PD that can appear years, sometimes decades, before the onset of the motor phenotype [1], [2], [3]. These symptoms can include hyposmia, disrupted sleep patterns, gastrointestinal disturbance, anxiety, depression, autonomic dysfunction, and impaired cognition [1], [2], [3]. Interestingly, cognitive dysfunction was recently identified as a prognostic factor for more severe onset of motor impairment and disability during the first five years following initial PD diagnosis [4], and while levodopa treatment is reasonably effective in mitigating motor symptoms, several studies have demonstrated that it has poor, or even negative, therapeutic value in treating cognitive symptoms in PD [5], [6], [7], [8], [9]. This disconnect suggests that distinct neuronal networks are involved in regulating motor versus cognitive symptoms. Unfortunately, the preclinical models of PD that are currently available to investigate the pathogenic mechanisms and explore novel therapeutic strategies are inadequate.

The classical preclinical models of PD involve the use of 6-hydroxydopamine (6OHDA) or 1-methyl 4-phenyl 1,2,3,6-tetrahydropyridine (MPTP) to destroy dopaminergic neurons. Also, genetic linkage studies in humans have led to the development of many transgenic mouse models of PD. Data using these models have provided insight into the pathogenic role of specific genes, such as alpha synuclein, LRRK2, Parkin within the context of familial forms of PD [10]. Mitochondrial dysfunction has emerged as a putative pathogenic point of convergence in the development of PD, based on both clinical observations and the aforementioned preclinical models [11]. As such, a new mouse model, called MitoPark, has been developed to investigate whether mitochondrial dysfunction in dopaminergic neurons causes parkinsonian pathology and phenotype [12]. The initial characterization of MitoPark mice has demonstrated a striking recapitulation of clinical PD pathogenesis. Importantly, MitoPark mice exhibit the cardinal features of PD, including adult onset of neurodegeneration, progressive decline in motor function, presence of intraneuronal inclusions (possibly a Lewy body equivalent), preferential death of dopaminergic neurons in the SN compared to the VTA and responsiveness to levodopa (L-DOPA) [11], [12], [13]. However, the development of non-motor, neuropsychiatric-like symptoms has not been characterized in the MitoPark mouse model.

Cognitive impairment represents one of the most prevalent and early developing non-motor symptoms of PD [14], [15]. In fact, cognitive dysfunction at the time of diagnosis appears to be an accurate predictor of how quickly motor impairment and disability occurs [4]. Here, we measured spatial learning and memory using the Barnes maze or novel object recognition in MitoPark mice prior to the onset of motor impairment or after a significant motor phenotype had developed. By twenty weeks of age, MitoPark mice exhibited a profound reduction in locomotor activity that precluded any assessment of their performance during cognitive testing. However, young, phenotypically normal MitoPark mice exhibited a significant delay in the acquisition (learning) phase of the test compared to littermate controls, but retention of the task once it was learned (memory) was not different between groups. Further analysis revealed that the delayed learning curve of young MitoPark mice was characterized by impaired within-day learning. Together these important new data indicate that the MitoPark mouse model of PD faithfully recapitulates the development of an important non-motor phenotype frequently observed in patients; thus, this model may represent an important preclinical resource to explore the underlying neuropathology of PD-associated cognitive dysfunction and test novel therapeutic approaches.

## Materials and Methods

### Animals and Husbandry

MitoPark (DAT^+/cre^-Tfam^loxP/loxP^) were previously generated [12], and we obtained breeding pairs from Dr. Nils-Göran Larsson (Karolinska Institutet, Stockholm, Sweden; now at Max Planck Institute for Biology of Aging, Cologne, Germany). To generate experimental mice and same-litter controls, MitoPark (on a C57BL6 background) mice were backcrossed to C57BL/6J (The Jackson Laboratory, Bar Harbor, ME), and offspring were selectively mated to generate double heterozygous males (DAT^+/cre^-Tfam^+/loxP^) and homozygous floxed Tfam females (DAT^+/+^-Tfam^loxP/loxP^). Offspring from this breeding combination will result in a 25% MitoPark (DAT*^+/cre^*; Tfam*^loxP/loxP^*) and 50% wild-type phenotype, littermate controls (DAT^+/+^-Tfam^+/loxP^ ^or loxP/loxP^). Mice were group-housed in same-sex, standard shoebox cages within a ventilated caging system with *ad libitum* access to food and water. Initial experiments were performed using both male and female mice, as our initial analysis of data probing for sex effects indicated that there was not an interaction between sex and genotype on any of the observed behavioral phenotypes. Therefore, to increase power within our experiments, the data were collapsed into two groups including both sexes prior to analysis. The room was maintained at 26°C on a 12-hr light/12 hr dark light cycle, (on at 07∶00). All animal care and use was carried out in accord with the Guide for the Care and Use of Laboratory Animals, 8^th^ edition (NRC) and approved by the Institutional Animal Care and Use Committee at the University of Texas Health Science Center at San Antonio.

### Genotyping

DNA was extracted from a tail snip by digestion in buffer containing 10 mM Tris-HCL,50 mM KCL and 0.1%Tween-20 with proteinase K solution (0.2 mg/ml), incubated at 55°C overnight. Samples were centrifuged at 15,000×g for 20 minutes, and supernatant was collected as DNA extract. Polymerase chain reaction (PCR) was performed on 0.5 µg of DNA. To identify the DAT^+/*cre*^ genotype, a multiplex PCR setting with two primer pairs was used. Forward primer sequence was 5′-CATGGAATTTCAGGTGCTTGG, and the reverse primer sequences were 5′-CATGAGGGTGGAGTTGGTCAG and 5′-CGCGAACATCTTCAGGTTCT, which enabled identification of heterozygous mice. For the Tfam*^loxp/loxp^* genotype, two primer pairs were also used. The forward primer sequence was 5′-CTGCCTTCCTCTAGCCCGGG, and the two reverse primer sequences were 5′-GTAACAGCAGACAACTTGTG and 5′-CTCTGAAGCACATGGTCAAT, which distinguished between heterozygous and homozygous mice. GoTaq® Green Master Mix (Promega, Madison, WI) was used for a 35 cycle PCR with the following settings: 95°C for 30 seconds, 55°C for 30 seconds and 72°C for 45 seconds. The PCR products were separated by electrophoresis on 2% agarose gels and were visualized under UV light after ethidium bromide staining. Two bands at 310 and 470 base pairs for DAT*^+/cre^* and one band at 437 base pairs for Tfam*^loxp/loxp^* should be detected from MitoPark mice. One band at 310 base pairs for DAT*^+/+^* (wildtype) and one band at 437 base pairs for Tfam^+/loxP or *loxp/loxp*^ should be detected from control littermates.

### Locomotor Assessment

Both horizontal and vertical locomotor activities were measured by the Opto-Max Activity Meter (Columbus Instruments, Columbus, Ohio, USA) according to the manufacturer’s protocol. Data were collected for 60 min and assessed at 10-min time intervals for locomotor activity.

### Barnes Maze (Acquisition)

Spatial learning and memory was measured in the Barnes maze as previously described [16] with minor modifications. Briefly, a commercially manufactured maze (91 cm diameter with 20×5 cm diameter holes around the perimeter) was used (Stoelting Co., Wood Dale, IL). All but one of the holes contained a false bottom, while the target hole led to an escape burrow below the maze surface. The location of the target hole was constant in relation to various visual cues placed on the walls of the testing room. Testing took place under fluorescent overhead lighting with a gentle breeze blown across the maze by a small, 15 cm diameter, office fan to stimulate mice to explore the maze. The acquisition period (learning phase) consisted of three daily trials taking place on four consecutive days with an inter-trial interval of 25 minutes. To begin each trial the test mouse was placed under an opaque start box in the center of the maze for 10 seconds. The box was removed, and the mouse was allowed to freely explore the maze for 3 minutes while a digital video camera recorded the trial from overhead. When the test mouse entered the burrow via the target hole, the hole was covered and the mouse left undisturbed for one minute. If the test mouse failed to enter the hole by the end of the trial, it was gently prodded into the burrow and left undisturbed for one minute. The maze was cleaned with 70% ethanol between mice. Ethovision XT tracking software (Noldus, Leesburg, VA) was used to determine the latency to find the target hole, path length traveled to find the target hole and locomotor velocity during the test. Sampling distribution (errors) of all holes was measured manually by a trained observer. Video records were also screened for the presence of common stereotypies, including over-grooming, rearing and excessive paw to mouth movements. No discernable difference between wild type or MitoPark mice was noted.

### Barnes Maze (Probe Trial)

Short term memory was tested in a single probe trial 24 hours after the final day four acquisition trial. Seven days after the first probe trial, a second probe trial was conducted to assess long-term memory. Each probe trial lasted 90 seconds. The burrow was removed from under the maze, and the target hole was fitted with an identical false bottom as the rest of the holes. The latency and path length traveled to find the correct target hole, velocity, and duration of the probe trial spent in proximity to the correct target hole were measured using Ethovision XT tracking software (Noldus). Sampling distribution (errors) was measured manually by a trained observer.

### Novel Object Recognition Task

To minimize the effects of stress, mice were transported to testing room and gently handled for two consecutive days. On the third and fourth day, mice were placed in the empty training/testing arena (40×40 cm) and allowed to habituate for 7 minutes each day. Training took place on the fifth day. Mice were placed in the corner of the arena containing two identical objects (black 50 ml conical tubes filled with glass beads) placed along the midpoint of each opposite wall. Mice were allowed to freely explore the objects for 7 minutes. Mice were returned to their home cages and housing room after training. Twenty four hours after the training session (day 6), the mice were placed in the same arena with one familiar object (black 50 ml conical from the training session) and one novel object (clear 50 ml conical tube filled with glass beads) that the mouse had never seen. The novel object was placed in exactly the same location as the familiar object from the training day. In all, the procedure took 6 days for each mouse (summarized in Table 1), and was performed under dim overhead white lighting. The time spent by each mouse investigating the familiar or novel objects was scored from video records by a blinded observer. A discrimination index was calculated using the formula; [time exploring novel object/(time spent exploring novel+time spent exploring familiar)]*100.

**Table 1:** Novel Object Recognition Protocol.

Day	Procedure
1	Transport to room, gentle handling
2	Transport to room, gentle handling
3	Transport to room, habituate to empty arena for 7 minutes
4	Transport to room, habituate to empty arena for 7 minutes
5	7 minute training with two identical objects
6	7 minute testing with one familiar and one novel object

### Statistics

Data were analyzed using GraphPad Prism 5.03 (LaJolla, CA) and are represented as the means ± SEM. Body weight, activity and Barnes maze data were analyzed by one factor (genotype) or two factor (genotype and age) analysis of variance (ANOVA) with repeated measures where appropriate. When the ANOVA revealed a significant interaction (p<0.05), post-hoc analysis using a Bonferroni posttest was performed to determine differences between means. Comparison of within-day learning curves was carried out by slope analysis. For slope analysis, straight lines were fitted to the acquisition curves between the three within day trials and compared using an *F*-ratio test. Analysis of the discrimination ratio in the novel object recognition test was performed using an upaired t-test, and to determine whether the sampling frequency at a given hole during the probe trial was significantly different from the theoretical random rate (dashed line) a one sample *t*-test was performed. Significant effects are denoted when p<0.05. All data were determined to represent normal distribution with equal variance.

## Results

### MitoPark Mice Exhibit a Progressive Loss of Body Weight and Motor Function

**Figure 1:**
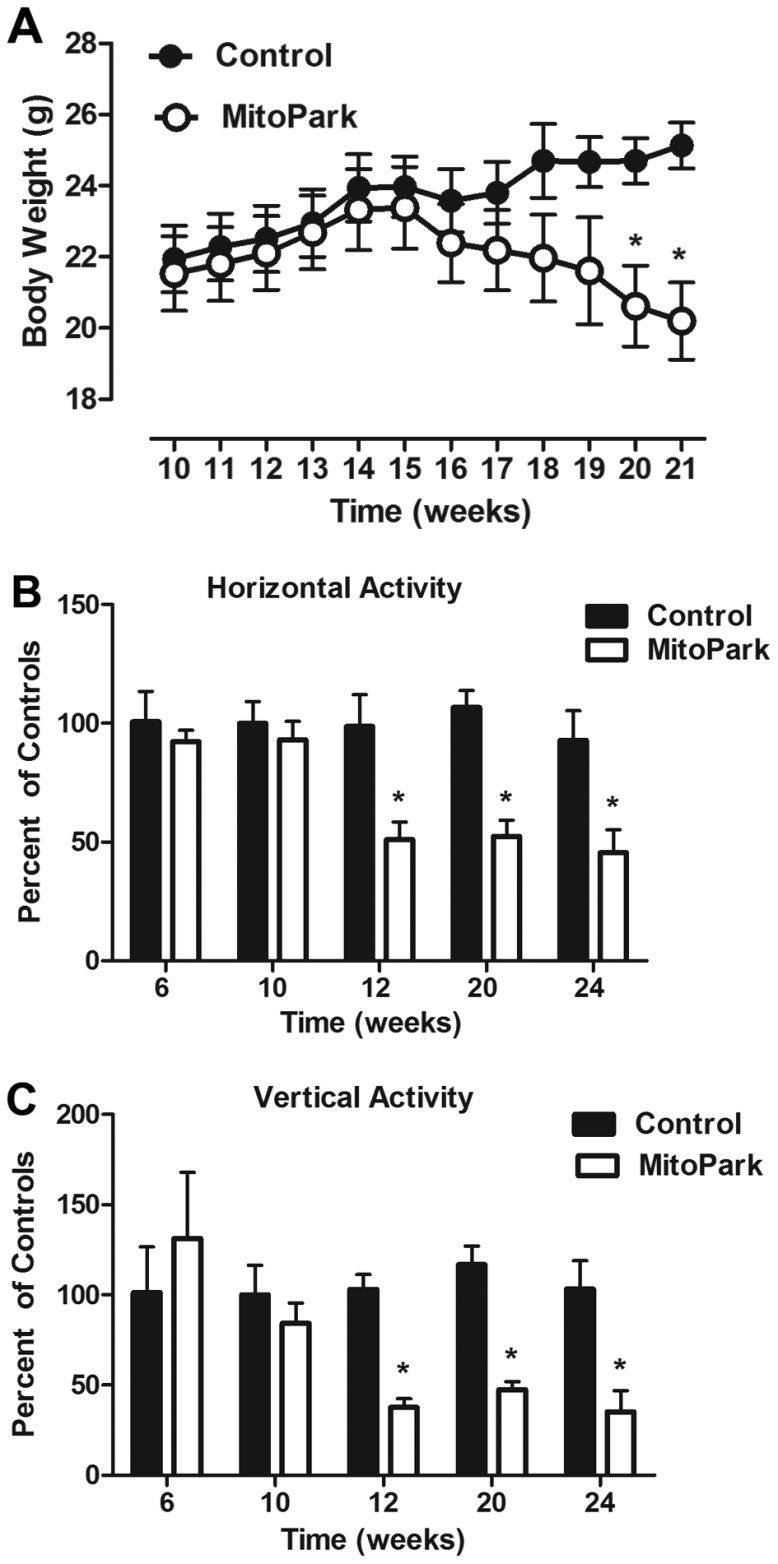
Progressive loss of body weight and motor function in MitoPark mice. (A) Body weights were recorded weekly in either MitoPark mice or their wild-type phenotype littermate controls. (B) Horizontal and (C) vertical locomotor activity were measured at the indicated times in both MitoPark mice or their wild-type phenotype littermate control mice. Data represent mean ± SEM, (n = 10 mice/group). Significant post hoc effect indicated (*p<0.05) for MitoPark versus same time point littermate controls.

A slow and progressive loss of motor function is a hallmark behavioral feature of MitoPark mice that lends validity to this new preclinical model [13]. Using body weight as an index of general health and vitality, as we have previously reported [17], [18], [19], MitoPark or littermate control mice were weighed weekly. While there was not a significant main effect of either time or genotype, a significant time×genotype interaction was apparent for body weight (**Figure 1A**, F_11,198_ = 2.19, p<0.02). No difference in body weight was apparent between genotypes until about 16 weeks of age, where the body weight curves began to diverge. Control mice displayed gradual and steady increases in their body weight, while MitoPark mice began to lose weight at 16 weeks. Post hoc analysis indicated that the difference in body weight was significant beginning at 20 week of age (p<0.05). Consistent with previous reports [13], a significant time×genotype interaction was apparent for both horizontal (**Figure 1B**, F_4,56_ = 2.73, p<0.05) and vertical (**Figure 1C**, F_4,56_ = 3.2, p<0.02) locomotor activity. In both cases, post hoc testing revealed a significant reduction in activity beginning at 12 weeks (p<0.05) in MitoPark mice when compared to same time point littermate control mice. Together, these data establish a clear timeline by which experiments exploring the development of non-motor symptoms in MitoPark mice can be carried out without the potential confound of reduced locomotor activity.

### A Mild Impairment in Spatial Learning Precedes the Overt Decline in Motor Function in MitoPark Mice

**Figure 2:**
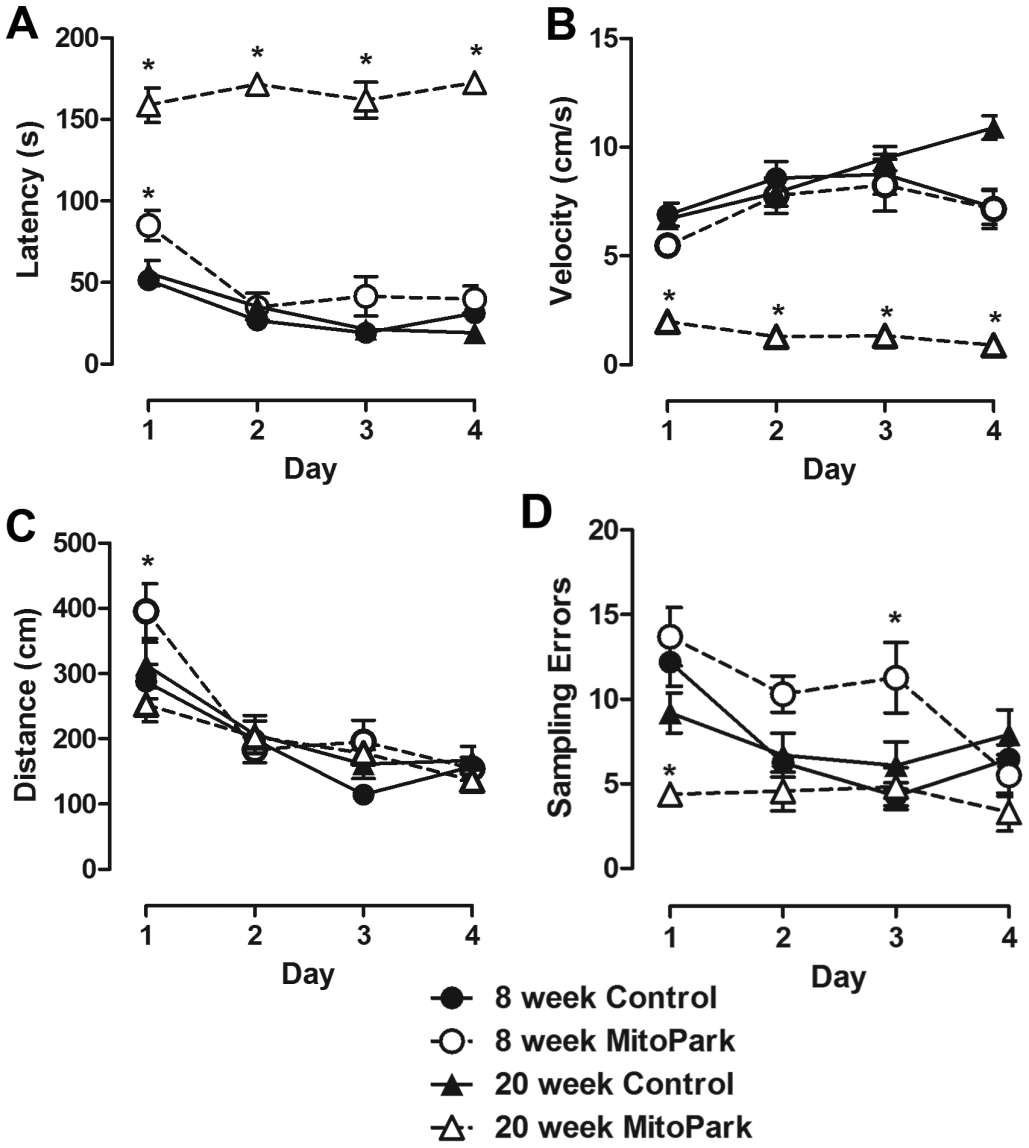
Profound motor impairment prohibits assessment of spatial learning in mature MitoPark mice. Spatial learning was assessed before (8 week old) or after (20 week old) the development of motor impairment in MitoPark mice or their age matched wild-type littermate controls. (A) Latency to locate the target hole, (B) velocity during the test, (C) distance traveled to locate the target hole and (D) the number of sampling errors on each of four successive training days was measured. Data represent the mean ± SEM of the daily averages for each mouse, (n = 8 mice/group). Significant post hoc effect indicated (*p<0.05) for MitoPark versus same age and day controls.

Mild cognitive impairment represents a highly prevalent and early developing symptom of patients with PD [14], [15], and few preclinical models recapitulate this clinical feature. To determine whether MitoPark mice develop cognitive dysfunction independent from deterioration of motor function, 8 week old (±1 week) or 20 week old (±2 week) MitoPark and littermate control mice were tested in the Barnes Maze. There was a significant main effect of time for each of the four variables measured; latency (F_3,132_ = 13.58, p<0.001), velocity (F_3,132_ = 8.06, p<0.001), distance (F_3,132_ = 36.97, p<0.001) and sampling errors (F_3,132_ = 9.24, p<0.001) (**Figures 2A–D**), and a significant main effect of genotype was apparent for only latency (F_3,132_ = 199.6, p<0.001), velocity (F_3,132_ = 49.45, p<0.001) and sampling errors (F_3,132_ = 10.91, p<0.001). Also, omnibus repeated measures ANOVA revealed a significant genotype by time interaction for all four latency (F_9,132_ = 4.01, p<0.001), velocity (F_9,132_ = 4.86, p<0.001), distance (F_9,132_ = 2.1, p<0.05) and sampling errors (F_9,132_ = 3.25, p<0.01). While post hoc testing indicated that MitoPark mice exhibited impaired performance during the test, profound motor deficits in the 20 week old MitoPark mice (**Figure 2B** **,** p<0.001 vs. same age littermate controls**)** precluded a clear interpretation regarding their cognitive performance. Interestingly, in 8-week-old mice, post hoc testing revealed deficits in learning that were not confounded by loss of motor function. Specifically, while the average latency to discover the target hole decreased in both 8-week-old MitoPark and control mice over the four day acquisition period, latency was significantly increased on the first day of acquisition (**Figure 2A**, p<.05) and tended to be increased on day 3 (p<0.10). In parallel to latency, post hoc testing indicated that the average distance traveled in locating the target hole was significantly increased on day one (**Figure 2C**, p<0.05). MitoPark mice also committed significantly more sampling errors on day 2 (trend; p<0.1) and day 3 (p<0.05) as compared to their littermate controls (**Figure 2D**). Importantly, these data were not confounded by differences in motor function, as average horizontal velocity during the test was not different between 8-week-old MitoPark and littermate controls on any day of testing (**Figure 2B**). Together, these data indicate that MitoPark mice have impaired spatial learning compared to littermate controls, and this cognitive deficit precedes the loss of motor function.

### MitoPark Exhibit Impaired within-day Spatial Learning on the Barnes Maze

**Figure 3:**
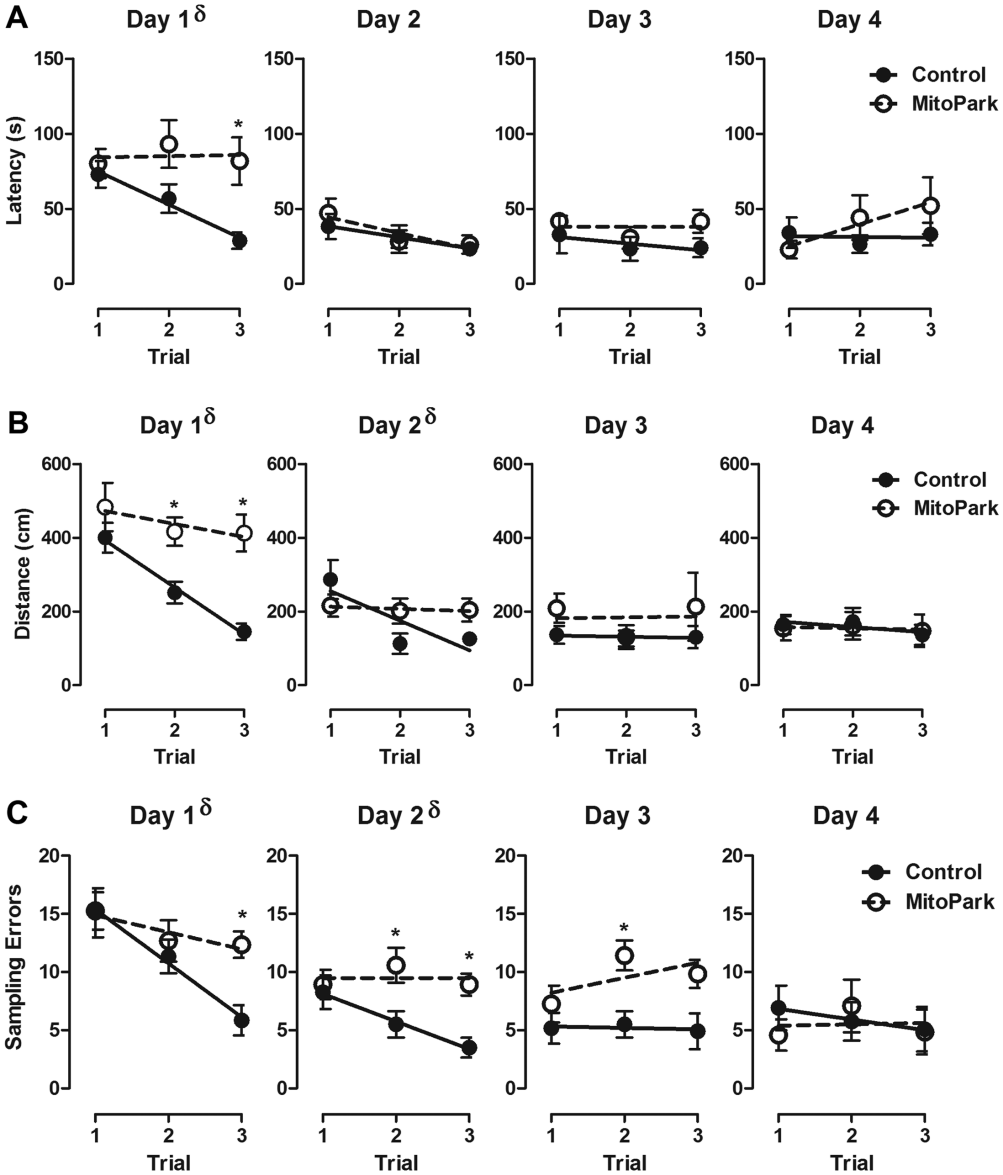
MitoPark mice exhibit impaired within-day spatial learning. Spatial learning in 8 week old MitoPark or wild-type phenotype littermate control mice within a given day was assessed by comparing performance across successive daily trials. (A) Latency to locate the target hole and (B) Distance traveled to locate the target hole measured for each of the three trials on four successive days. (C) The number of sampling errors recorded for each trial measured over the four successive training days. Data represent mean ± SEM, (n = 8 mice/group). Significant post hoc effect indicated (*p<0.05) for MitoPark and same day and trial littermate control. § p<0.05 difference in learning curve (slope) between MitoPark and littermate control mice on a given day.

To further characterize the deficits in spatial learning exhibited by MitoPark mice, within-day learning was assessed by two-way repeated measures ANOVA with trial as the within-subject factor and genotype as the between subject factor as previously described [20]. A significant trial×genotype interaction was apparent for latency (**Figure 3A**; F_11,242_ = 2.45, p<0.05), distance (**Figure 3B**; F_11,242_ = 5.31, p<0.05) and sampling errors (**Figure 3C**; F_11,242_ = 5.05, p<0.05). Velocity was not significantly different between 8-week-old MitoPark and littermate controls. Post-hoc analysis of the revealed that even after three trials, the latency for MitoPark mice to locate the target hole was significantly higher than littermate controls (**Figure 3A**; p<0.05), and the distance traveled to located the target hole was higher for MitoPark mice during both the second and the third trials on day one (**Figure 3B**; p<0.05). Additionally, MitoPark mice committed significantly more sampling errors on multiple trials across the first three days of acquisition testing (**Figure 3C**; post hocs p<0.05). To confirm whether the rate of learning (slope) within a test day was different between MitoPark and littermate controls, a linear best fit line was applied to each day’s data. MitoPark mice failed to exhibit any reduction in their latency to locate the target hole, with a day one learning curve (slope) not significantly different from zero (p = 0.94) but significantly different from littermate controls (**Figure 3A**; F_1,68_ = 4.02, p<0.05). The same pattern was apparent for distance to locate the target hole on days one and two, where the MitoPark learning curves (slopes) were not different from zero (day 1, p = 0.35; day 2, p = 0.79) but were significantly different from littermate controls (**Figure 3B**; day 1, F_1,68_ = 4.6, p<0.05; day 2, F_1,68_ = 4.61, p<0.05). When analyzing the sampling errors between the successive trials of a given day, MitoPark mice, again, fail to demonstrate any within-day improvement on the task. Not only are their learning curves (slopes) flat each of the four days, but also they are significantly different than littermate controls on both the first and second day of testing (**Figure 3C**; day 1, F_1,68_ = 4.02, p<0.05; day 2, F_1,68_ = 3.86, p<0.05). Together, these data indicate that the spatial learning deficit apparent in 8 week old MitoPark mice is characterized by impaired learning between the successive within day trials.

### Probe Trial Performance was not Different between MitoPark or Control Mice

**Figure 4:**
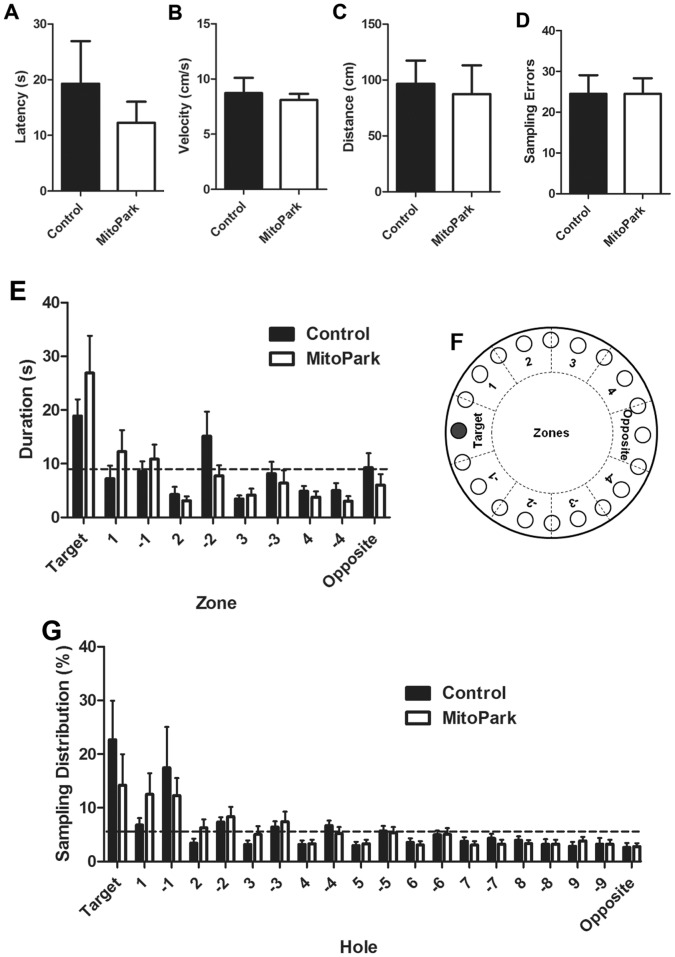
Behavior was assessed during a single probe trial 24 hours after the final day 4 training trial. The (A) latency to locate the correct target hole, (B) velocity during the trial, (C) distance traveled to locate the target hole and (D) the total number of sampling errors committed during the trial were measured in 8 week old MitoPark or wild-type phenotype littermate control mice. (E) The duration of time spent in each of ten identical virtual zones (schematic in F) around the perimeter of the maze was recorded, and (F) the distribution of sampling errors was recorded. Dashed lines represent the theoretical threshold if exploratory behavior were random. Data represent mean ± SEM, (n = 8 mice/group).

**Figure 5:**
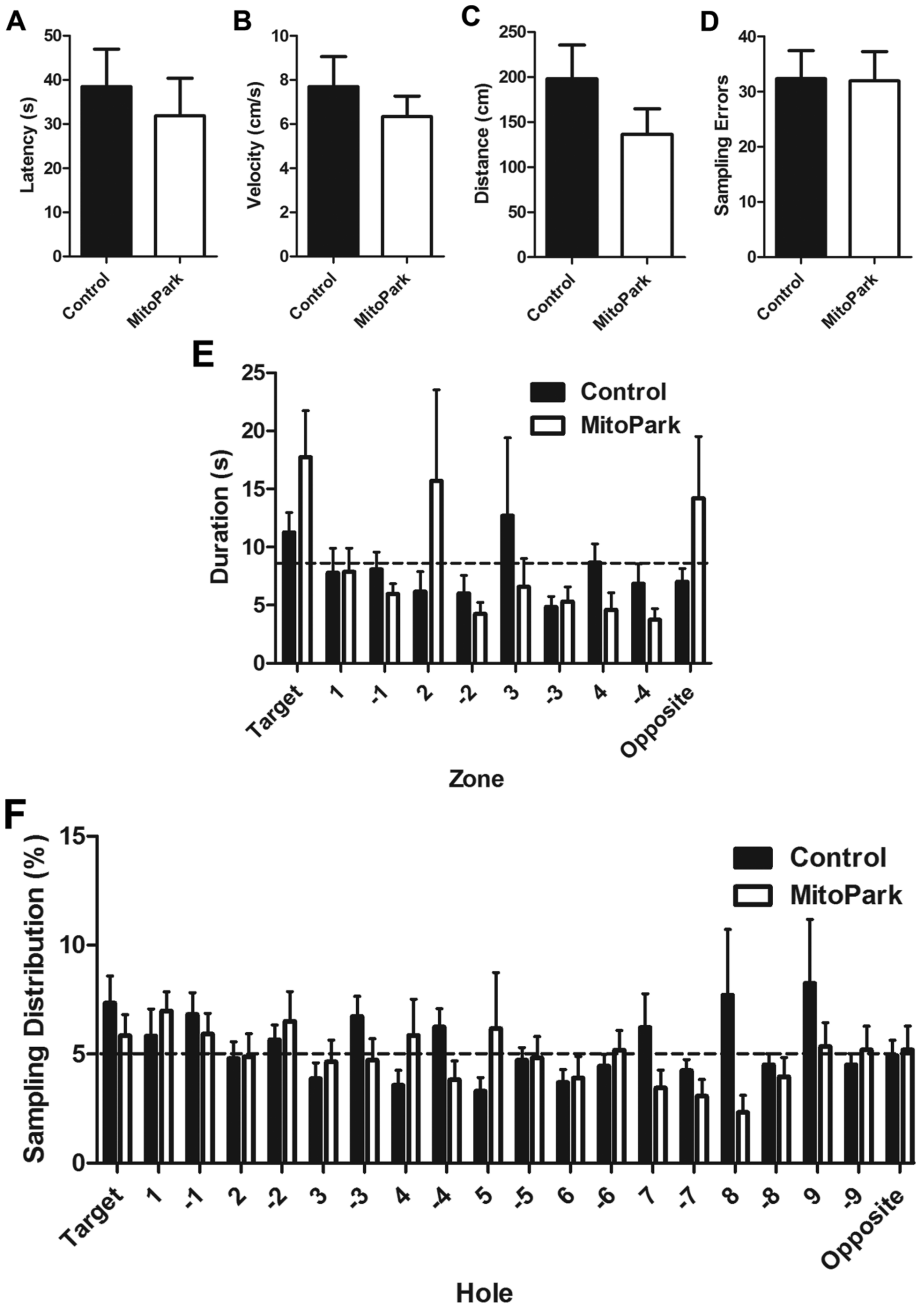
A second probe trial was conducted seven days after the first to assess memory of the previously learned task. The (A) latency to locate the correct target hole, (B) velocity during the trial, (C) distance traveled to locate the target hole and (D) the total number of sampling errors committed during the trial were measured in 8 week old MitoPark or wild-type phenotype littermate control mice. (E) The duration of time spent in each of ten identical virtual zones (schematic in Figure 5F) around the perimeter of the maze was recorded, and (F) the distribution of sampling errors was recorded. Dashed lines represent the theoretical threshold if exploratory behavior were random. Data represent mean ± SEM, (n = 8 mice/group).

Although MitoPark mice display deficits in learning, by the fourth day, performance on the Barnes maze was not different between genotypes (**Figures 2** **and** **3**). As an initial test of memory, a probe trial was conducted on the fifth day of testing (24 hours after the final acquisition session). There was no significant difference in latency to locate the target hole (**Figure 4A**), velocity during the test (**Figure 4B**), distance traveled to locate the target hole (**Figure 4C**) or the average number of sampling errors during the first probe trial (**Figure 4D**) between MitoPark mice or their littermate control mice. We also analyzed the duration of time spent (**Figure 4E**) in ten identical virtual zones (illustrated in **Figure 4F**) and the sampling distribution (**Figure 4G**) during the probe trial. There was not a significant difference between MitoPark and littermate control mice for either variable. Both MitoPark and control mice spent significantly more time in the zone containing the target hole than would be predicted if behavior were at random as indicated by the dashed line (**Figure 4E**; p<0.01 and p<0.05 for MitoPark and control, respectively). The duration spent in all other zones for both genotypes were either no different or lower than the random behavior threshold. Similarly, analysis of the sampling distribution during the probe trial indicated that both MitoPark and control mice investigated the target hole and the immediately adjacent holes at a higher frequency than more distal holes (**Figure 4G**). There was not a significant difference between genotypes. A second probe trial was conducted seven days after the first probe trial. Similar to the first probe trial, no significant difference in latency to locate the target hole (**Figure 5A**), velocity during the test (**Figure 5B**), distance traveled to locate the target hole (**Figure 5C**) or the average number of sampling errors during the probe trial (**Figure 5D**) between MitoPark mice or their littermate control mice. However there was a tendency for all mice to take longer, travel further and make more errors in locating the target hole. Analysis of the duration spent within the target zone during the probe trail revealed that neither MitoPark nor littermate control mice spent more time than the predicted random duration in any zone (**Figure 5E**). Consistent with this observation, the sampling distribution of both MitoPark and littermate control mice during the second probe trial reflected a completely random sampling pattern (**Figure 5F**). Together, these data indicate that memory, as assessed by probe trial performance is not disrupted in 8-week-old MitoPark mice.

### Novel Object Recognition is Impaired in MitoPark Mice

**Figure 6:**
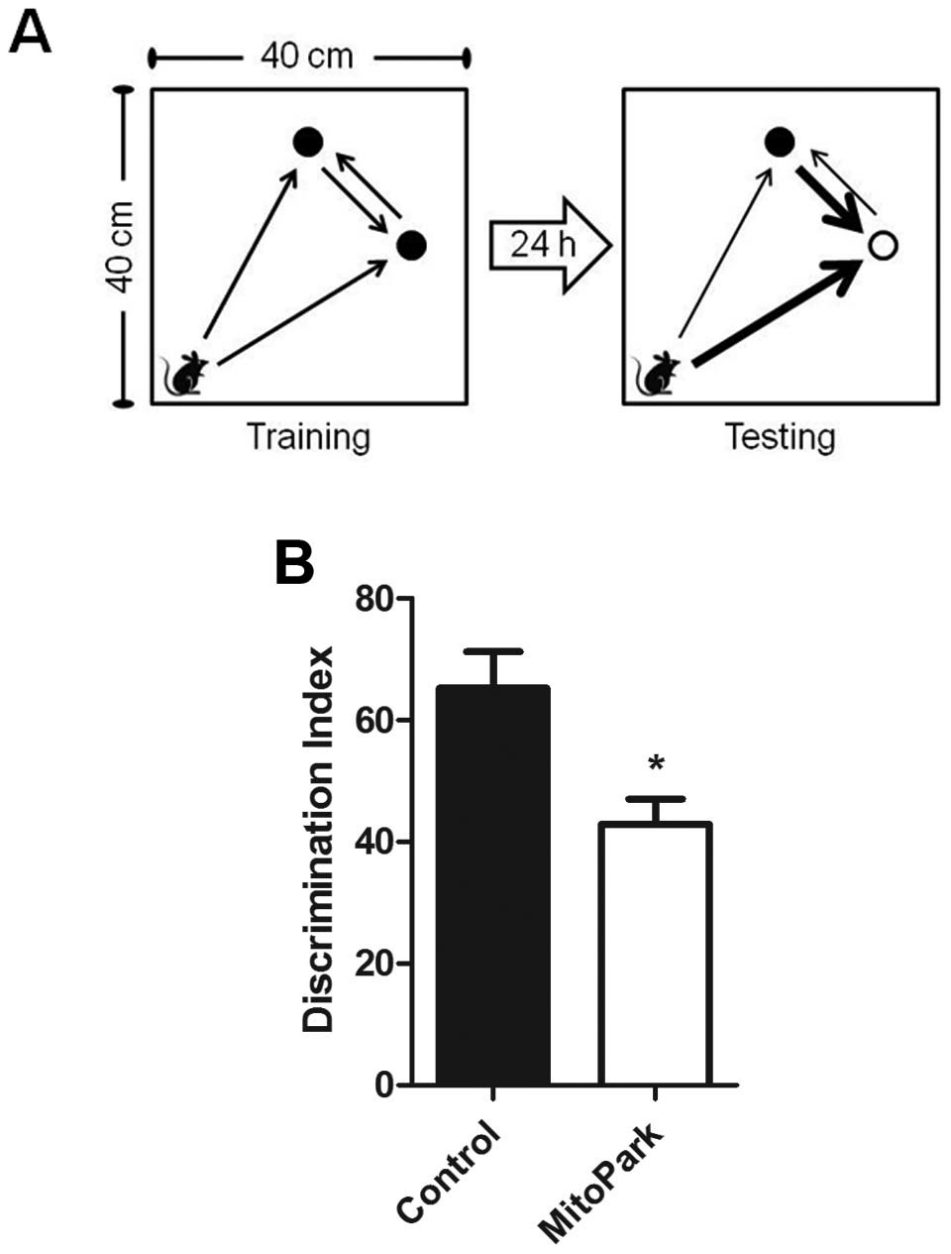
Novel object recognition is impaired in MitoPark mice. (A) A Schematic illustrating the arena setup for both training and testing phases of the task. (B) 24 h after exposure to two identical objects in an open arena, the discrimination index was assessed by measuring the duration of time spent exploring the novel object versus the total time spent exploring both objects was determined. Data represent mean ± SEM, (n = 8 mice/group). *p<0.01 for MitoPark versus littermate controls.

To confirm the development of early cognitive dysfunction, preceding motor impairment, in the MitoPark mice, a group of 8 week old MitoPark mice or their WT control littermates were tested in the novel object recognition task. As illustrated in **Figure 6A**, mice were exposed to two identical objects followed by a training-testing interval of 24h. In this paradigm, previous reports indicate that C57BL6 mice exhibit a significant preference for the novel object. Here, the discrimination index of control mice was, indeed, significantly higher than 50% (**Figure 6B**; *t_8_* = 2.52, p<0.05). Interestingly, MitoPark mice displayed a significantly lower discrimination index as compared to littermate control mice (**Figure 6B**; *t*_16_ = 3.06, p<0.01) that was not significantly different from 50%, which is the level indicative of no preference. Together, these data identify a general impairment in cognitive function in the MitoPark mouse that is independent from the progressive parkinsonian-like loss of motor function.

## Discussion

To the best of our knowledge, the present study is the first to examine the development of non-motor symptoms in the MitoPark mouse model of PD. This transgenic mouse model was based on evidence of mitochondrial dysfunction in PD. The mitochondrial transcription factor Tfam was selectively deleted in dopaminergic neurons, which leads to progressive respiratory chain deficiency and eventual cell death [12]. In terms of cellular pathology and motor function, MitoPark mice recapitulate the clinical features of sporadic PD very well. However, patients with PD suffer from a wide range of non-motor symptoms; cognitive impairment being chief among them. Whether MitoPark mice also show non-motor PD symptoms, particularly cognitive impairment, is the focus of this study.

Here, we first confirmed, in our hands, that MitoPark mice develop a progressive parkinsonian-like deterioration of motor function, while wild-type phenotype littermate control mice do not. Consistent with previous reports [13], we observed the first significant reduction in locomotor activity at 12 weeks, and mice exhibited a continued decline over the next 4–5 months. By 20 weeks of age, MitoPark mice had significantly reduced body weight, and their reduction in locomotor activity was severe enough that cognitive function could not be assessed from the data we collected. They simply failed to adequately perform the test. Interestingly, we observed a clear deficit in the ability of young, 8 week old, MitoPark mice to locate the target escape hole during the acquisition (learning) period of the Barnes maze, although by the fourth day of acquisition training, MitoPark mice performed similar to controls. This relatively mild cognitive impairment was characterized by a striking absence of within day learning. Particularly, during the first two days of acquisition testing, when control mice exhibit marked improvement across the three daily trials, MitoPark mice failed to improve between trials. Using a probe trial as a proxy of memory revealed no difference between groups of mice. Interestingly, 8-week old MitoPark mice also exhibited a deficit in novel object recognition, which relies on a neural circuit distinct from the Barnes maze. In all, these data are the first to establish the presence of a clinically relevant non-motor phenotype in the MitoPark model of PD.

Many preclinical models of PD exhibit a loss of motor function, but a recent study by Galter et. al. noted that the progression of motor symptoms in the MitoPark mice may uniquely resemble of the onset of symptoms in PD patients [13]. In their study, vertical movement decreased sooner than horizontal movement in MitoPark mice. The authors speculated that particular behavioral profile might resemble the onset of axial/postural instability, which is an early occurrence in the progression of motor symptoms in patients with PD. Further, their study demonstrated that MitoPark mice displayed a double-peaked locomotor response to high doses of L-DOPA, which might resemble the “on-off” phenomenon of PD [13]. Our data indicate that both vertical and horizontal locomotor activity were significantly reduced at 12 weeks, which was the same time point used by Galter et. al. to dissociate the two behaviors. They reported a significant 65% reduction in vertical activity and a 25% reduction in horizontal activity that failed to reach significance. Closer analysis of our data revealed that vertical activity of MitoPark mice, when compared directly to horizontal activity at the 12 week time point, was significantly lower (horizontal % of controls- 58.37±6.4 versus vertical % of controls- 42.02±6.9, p<0.002). In all, the two data sets support the same conclusion that vertical activity may be more significantly impaired earlier in the progression of motor symptoms in MitoPark mice. Whether this can be taken as a preclinical reflection of axial/postural instability observed in patients with PD remains speculative.

The present study did not examine the effects of L-DOPA treatment on the progression of motor symptoms, as this has already been thoroughly established [13]. However, cognitive impairment in PD is not particularly responsive to L-DOPA treatment [21]. In fact, increased homocysteine levels that occur due to L-DOPA administration in PD patients has been implicated as a causative factor in cognitive dysfunction and dementia [8], [9]. A recent study in healthy young volunteers found that L-DOPA administration impaired visual-spatial cognitive performance [7], and in chronic MPTP treated Cynomolgus monkeys, doses of L-DOPA that were therapeutically effective in improving motor function actually precipitated additional cognitive deficits [5]. It should be noted that several laboratories have demonstrated cognitive disruption in the MPTP (dopaminergic neurotoxin) model of PD, but a limitation of the MPTP model is that these changes typically coincide with loss of motor function, which can confound interpretation of behavioral testing in rodents. Also, in mice, MPTP resulted in a reduction in calcium/calmodulin dependent protein kinase II activity concomitant with impaired long term potentiation in the CA1 hippocampal subregion [22], so MPTP might precipitate functional changes outside of the dopamine system to influence cognitive function. In contrast, the primary pathogenic defect in MitoPark mice is restricted to the dopaminergic system, and cognitive impairment appears to be associated with the progressive loss of function in dopaminergic neurons, not cell death; thus, these mice may provide a valuable model in which new therapeutic strategies to treat PD-associated cognitive impairment can be explored. Our data indicate that cognitive disruption begins at least four weeks prior to the progressive loss of motor function, so future experiments to determine whether L-DOPA restores cognitive function independent of motor function in MitoPark mice will be informative.

We utilized the Barnes maze and the novel object recognition task to assess cognitive function in the present study. These two cognitive tasks are primarily regulated by distinct brain regions. The Barnes maze is a hippocampal-dependent, spatial learning and memory task often regarded as a dry version of the more well-known Morris water maze. Novel object recognition is dependent on the perirhinal and entorhinal cortices [23], [24]. Interestingly, distinct subsets of VTA dopamine neurons project to these regions, and while we did not measure performance in prefrontal-cortical dependent cognition, our data suggest that the MitoPark mouse may be a useful tool to begin dissecting the role of dopamine regulation on various domains of cognitive function. There were several important considerations in selecting which cognitive test to use. Foremost, the Barnes maze is much less dependent on motor function than the Morris water maze, where mice are required to swim in order to locate a hidden escape platform. Without the aversive stimuli of being placed in a pool of water, the Barnes maze allows mice to freely explore the maze with minimal physical demand and without the potential confound of acute psychological stress. The Barnes maze also utilizes an ethologically more relevant approach than the water maze. As opposed to rats, mice are not strong swimmers; thus requiring mice to locate a target hole leading to a dark escape burrow below the maze is more consistent with the natural burrowing behavior of this nocturnal species. Similarly, the novel object recognition task relies on the mouse’s inherent curiosity to explore novel elements within their environment without the presence of any acute stressors. It is also possible that subtle changes in motor function, not detected by our methodology, are present at 8 weeks, so minimizing the requisite physical demands of the cognitive task was preferable.

Although not the focus of the present study, the MitoPark model may also provide a unique opportunity to explore the mechanisms by which the dopaminergic system regulates cognitive function. Not only are the mechanisms by which cognitive impairment occurs in PD poorly understood, but also we know surprisingly little about the role of the dopaminergic system on cognitive processes in general. MitoPark mice exhibit a preferential loss of dopaminergic neurons feeding into the nigrostriatal circuit relative to the ventral tegmental area (VTA), which constitutes the mesocortical and mesolimbic circuits. Ekstrand et. al. showed that tyrosine hydroxylase (TH) immunoreactivity in the substantial nigra was reduced approximately 35% relative to controls at 12 weeks of age, while TH staining in the VTA was similar to controls (∼90%) at the same time [12]. By 4–6 months, TH immunoreactivity was less than 50% of controls, and this corresponded with a marked reduction in dopamine levels [12]. In this study, we observed functional cognitive deficits appearing by 8 weeks of age in MitoPark mice. While viability (TH staining or DA levels) of dopamine cells in the MitoPark mice has not been measured at 8 weeks of age, Galter et. al. measured the decline in dopamine concentrations across a timeline from 6 to 40 weeks [13]. Specifically, dopamine levels were not reduced in either the striatum, olfactory bulb or cortex at 6 weeks, but striatal levels began to decline by 12 weeks. Dopamine levels in the cortex did not significantly decline until 24 weeks, while olfactory bulb dopamine loss was not apparent until 40 weeks. Together, their data are consistent with the progressive nature by which functional motor deficits appear in the MitoPark mice, but they also illustrate the notion that distinct brain regions relevant to functional behavioral outcomes are differentially impacted. Our data would suggest that death of dopamine cells is not necessary to drive functional deficits; rather, a progressive loss in neuronal function that precedes cell death is sufficient. In fact Good et al. identified electrophysiological changes in the nigrostriatal system as early as 6 weeks, which would correspond with the time in which we detected cognitive disruption [25]. While little is known about the mechanistic role, direct or indirect, of the substantia nigra on learning processes, Cunha et al. [26] demonstrated deficits in spatial learning, using a constant start location paradigm, in rats with a lesion of the substantia nigra pars compacta. However lesioned rats performed equal to controls if a variable start location paradigm was employed. Repeated testing in the constant start paradigm is generally thought to reflect stimulus-response habit learning or learning to repeat identical movements. It is worth noting that the Barnes maze protocol that we followed used a constant start location, so the absence of within day learning that we observed in the MitoPark mice could reflect early dysfunction within the substantia nigra or the inability of the mice to effectively utilize a search strategy. Dopaminergic cells from the substantia nigra could also modulate cortical and/or hippocampal processes indirectly via innervations of the thalamus. Additional tests are required to assess whether MitoPark mice exhibit deficits in frontal cortical mediated cognitive function.

In conclusion, the present data establish that the MitoPark mouse model of PD displays an important and clinically relevant non-motor symptom, cognitive impairment. A disruption in both spatial learning and novel object recognition was apparent well before the progressive loss of motor function, which is quite consistent with the etiology of PD in patients. Therefore, MitoPark mice may provide a unique opportunity to study mechanisms by which the dopaminergic system regulates behavior and investigate novel therapeutic strategies to treat both motor and non-motor symptoms of PD.

## References

[1] Chaudhuri KR, Healy DG, Schapira AH (2006) Non-motor symptoms of Parkinson’s disease: diagnosis and management. Lancet Neurol 5: 235–245. 16488379 10.1016/S1474-4422(06)70373-8

[2] Ziemssen T, Reichmann H (2007) Non-motor dysfunction in Parkinson’s disease. Parkinsonism Relat Disord 13: 323–332. 17349813 10.1016/j.parkreldis.2006.12.014

[3] Shulman LM, Taback RL, Bean J, Weiner WJ (2001) Comorbidity of the nonmotor symptoms of Parkinson’s disease. Mov Disord 16: 507–510. 11391746 10.1002/mds.1099

[4] Velseboer DC, Broeders M, Post B, van Geloven N, Speelman JD, et al (2013) Prognostic factors of motor impairment, disability, and quality of life in newly diagnosed PD. Neurology 80: 627–633. 23345637 10.1212/WNL.0b013e318281cc99

[5] Schneider JS, Pioli EY, Jianzhong Y, Li Q, Bezard E (2012) Levodopa improves motor deficits but can further disrupt cognition in a macaque parkinson model. Mov Disord. 10.1002/mds.25258 23238827

[6] Kulisevsky J (2000) Role of dopamine in learning and memory: implications for the treatment of cognitive dysfunction in patients with Parkinson’s disease. Drugs Aging 16: 365–379. 10917074 10.2165/00002512-200016050-00006

[7] Onur OA, Piefke M, Lie CH, Thiel CM, Fink GR (2011) Modulatory effects of levodopa on cognitive control in young but not in older subjects: a pharmacological fMRI study. J Cogn Neurosci 23: 2797–2810. 21254797 10.1162/jocn.2011.21603

[8] Zoccolella S, Lamberti SV, Iliceto G, Santamato A, Lamberti P, et al (2010) Hyperhomocysteinemia in L-dopa treated patients with Parkinson’s disease: potential implications in cognitive dysfunction and dementia? Curr Med Chem 17: 3253–3261. 20666719 10.2174/092986710792232012

[9] Zoccolella S, dell’Aquila C, Abruzzese G, Antonini A, Bonuccelli U, et al (2009) Hyperhomocysteinemia in levodopa-treated patients with Parkinson’s disease dementia. Mov Disord 24: 1028–1033. 19353704 10.1002/mds.22511

[10] Harvey BK, Wang Y, Hoffer BJ (2008) Transgenic rodent models of Parkinson’s disease. Acta Neurochir Suppl 101: 89–92. 10.1007/978-3-211-78205-7_15 PMC2613245 18642640

[11] Ekstrand MI, Galter D (2009) The MitoPark Mouse - an animal model of Parkinson’s disease with impaired respiratory chain function in dopamine neurons. Parkinsonism Relat Disord 15 Suppl 3S185–188. 20082987 10.1016/S1353-8020(09)70811-9

[12] Ekstrand MI, Terzioglu M, Galter D, Zhu S, Hofstetter C, et al (2007) Progressive parkinsonism in mice with respiratory-chain-deficient dopamine neurons. Proc Natl Acad Sci U S A 104: 1325–1330. 17227870 10.1073/pnas.0605208103 PMC1783140

[13] Galter D, Pernold K, Yoshitake T, Lindqvist E, Hoffer B, et al (2010) MitoPark mice mirror the slow progression of key symptoms and L-DOPA response in Parkinson’s disease. Genes Brain Behav 9: 173–181. 20002202 10.1111/j.1601-183X.2009.00542.x PMC4154513

[14] Kim JS, Oh YS, Lee KS, Kim YI, Yang DW, et al (2012) Association of cognitive dysfunction with neurocirculatory abnormalities in early Parkinson disease. Neurology 79: 1323–1331. 22972639 10.1212/WNL.0b013e31826c1acd PMC3448741

[15] Uc EY, McDermott MP, Marder KS, Anderson SW, Litvan I, et al (2009) Incidence of and risk factors for cognitive impairment in an early Parkinson disease clinical trial cohort. Neurology 73: 1469–1477. 19884574 10.1212/WNL.0b013e3181bf992f PMC2779004

[16] Lehmann ML, Brachman RA, Listwak SJ, Herkenham M (2010) NF-kappaB activity affects learning in aversive tasks: possible actions via modulation of the stress axis. Brain Behav Immun 24: 1008–1017. 20399847 10.1016/j.bbi.2010.04.005 PMC2897969

[17] Kelley KW, O’Connor JC, Lawson MA, Dantzer R, Rodriguez-Zas SL, et al.. (2013) Aging leads to prolonged duration of inflammation-induced depression-like behavior caused by Bacillus Calmette-Guerin. Brain Behav Immun. 10.1016/j.bbi.2013.02.003 PMC3686980 23454036

[18] O’Connor JC, Andre C, Wang Y, Lawson MA, Szegedi SS, et al (2009) Interferon-gamma and tumor necrosis factor-alpha mediate the upregulation of indoleamine 2,3-dioxygenase and the induction of depressive-like behavior in mice in response to bacillus Calmette-Guerin. J Neurosci 29: 4200–4209. 19339614 10.1523/JNEUROSCI.5032-08.2009 PMC2835569

[19] O’Connor JC, Lawson MA, Andre C, Briley EM, Szegedi SS, et al (2009) Induction of IDO by bacille Calmette-Guerin is responsible for development of murine depressive-like behavior. J Immunol 182: 3202–3212. 19234218 10.4049/jimmunol.0802722 PMC2664258

[20] Walker JM, Fowler SW, Miller DK, Sun AY, Weisman GA, et al (2011) Spatial learning and memory impairment and increased locomotion in a transgenic amyloid precursor protein mouse model of Alzheimer’s disease. Behav Brain Res 222: 169–175. 21443906 10.1016/j.bbr.2011.03.049

[21] McNamara P, Durso R (2006) Neuropharmacological treatment of mental dysfunction in Parkinson’s disease. Behav Neurol 17: 43–51. 16720959 10.1155/2006/138263 PMC5471533

[22] Moriguchi S, Yabuki Y, Fukunaga K (2012) Reduced calcium/calmodulin-dependent protein kinase II activity in the hippocampus is associated with impaired cognitive function in MPTP-treated mice. J Neurochem 120: 541–551. 22136399 10.1111/j.1471-4159.2011.07608.x

[23] Aggleton JP, Brown MW, Albasser MM (2012) Contrasting brain activity patterns for item recognition memory and associative recognition memory: insights from immediate-early gene functional imaging. Neuropsychologia 50: 3141–3155. 22634248 10.1016/j.neuropsychologia.2012.05.018

[24] Antunes M, Biala G (2012) The novel object recognition memory: neurobiology, test procedure, and its modifications. Cogn Process 13: 93–110. 22160349 10.1007/s10339-011-0430-z PMC3332351

[25] Good CH, Hoffman AF, Hoffer BJ, Chefer VI, Shippenberg TS, et al (2011) Impaired nigrostriatal function precedes behavioral deficits in a genetic mitochondrial model of Parkinson’s disease. FASEB J 25: 1333–1344. 21233488 10.1096/fj.10-173625 PMC3058704

[26] Da Cunha C, Silva MH, Wietzikoski S, Wietzikoski EC, Ferro MM, et al (2006) Place learning strategy of substantia nigra pars compacta-lesioned rats. Behav Neurosci 120: 1279–1284. 17201473 10.1037/0735-7044.120.6.1279

